# Electrical stimulation: a novel adjunct therapy for diabetic foot ulcers

**DOI:** 10.3389/fcdhc.2025.1682871

**Published:** 2026-01-07

**Authors:** Yuanjun Liu, Xiaoyu Liu, Jin Lu, Yunnan Jiang, Jian Wu

**Affiliations:** 1 The First Clinical School of Medicine, Gansu University of Chinese Medicine, Lanzhou, Gansu, China; 2 Burns Surgery Second Ward, Gansu Provincial Hospital, Lanzhou, Gansu, China

**Keywords:** diabetic foot ulcers, direct current stimulation, electrical stimulation, pulsed electric current, wound healing

## Abstract

Diabetic foot ulcer (DFU) is one of the most severe complications of diabetes; its healing is typically protracted and marked by a high rate of recurrence. In recent years, electrical-stimulation (ES) therapy has emerged as a novel adjunct to conventional approaches such as debridement, negative-pressure wound therapy, and moist dressings. By applying an exogenous electric field that mimics the skin’s endogenous wound currents, ES provides directional cues for cells and signaling molecules involved in repair, guiding them toward the wound bed. The external field reconstructs the bioelectric landscape, inducing oriented migration and proliferation of keratinocytes, fibroblasts, and endothelial cells, while up-regulating factors such as HIF-1α and VEGF to relieve local ischemia and promote neovascularization. Cathodal currents can also dampen the inflammatory cascade and facilitate the shift of macrophages from the M1 to the pro-healing M2 phenotype. The advent of nanogenerators, conductive hydrogels, and wireless “smart” bandages is gradually freeing ES from hard-wired leads, paving the way for home-based, closed-loop wound care. This review summarizes the latest mechanistic insights and device innovations, providing a reference for future clinical optimization and multicenter trials.

## 1 Introduction

Diabetes has become a global public-health challenge. In 2021, an estimated 537 million people were living with diabetes worldwide, more than 90% of whom had type 2 disease; this figure is projected to exceed 700 million by 2045 if effective control measures are not implemented (1). Diabetic foot ulcer (DFU) is among the most devastating complications of diabetes, with a lifetime incidence of 19 – 34%, a 3 – 5-year recurrence rate of roughly 65%, an amputation rate of about 20%, and a 5-year mortality of 50 – 70% (2). A systematic review covering more than 120–000 patients from 16 countries reported 5- and 10-year survival rates of only 50.9% and 23.1%, respectively, among individuals with DFU (3).

In current wound management, debridement, moist dressings and off-loading remain the cornerstones of therapy; nevertheless, overall healing rates remain below 60%, and mean healing time often extends over several months, rendering non-healing wounds a routine clinical challenge (4). Although negative-pressure wound therapy can improve perfusion and shorten the time to skin-graft readiness, its use is limited by device cost and patient adherence (5). Cutting-edge approaches such as nanocarriers and biomimetic dressings are still in the early clinical phase, and data on their efficacy and safety remain insufficient (6).

In this context, electrical stimulation has attracted attention because it can enhance the proliferation and migration of wound-healing–related cells (fibroblasts, endothelial cells, and keratinocytes), modulate inflammatory responses (e.g., interleukin-1), and improve local perfusion (7, 8). ES has been widely applied to chronic, hard-to-heal wounds such as pressure ulcers (9) and venous leg ulcers (10), and has even been used in plastic surgery to treat mandibular hypoplasia (11). Early randomized trials suggest that ES combined with ultrasound accelerates the closure of “refractory” DFU without serious adverse events; however, sample sizes were small and intervention parameters heterogeneous, leaving the evidence base inconclusive (12). A comprehensive appraisal of current evidence on ES for DFU is therefore clinically meaningful, as it may help reduce amputation rates and refine wound-care strategies.

## 2 Mechanism

### 2.1 Endogenous transepithelial potential and wound currents

**Figure 1:**
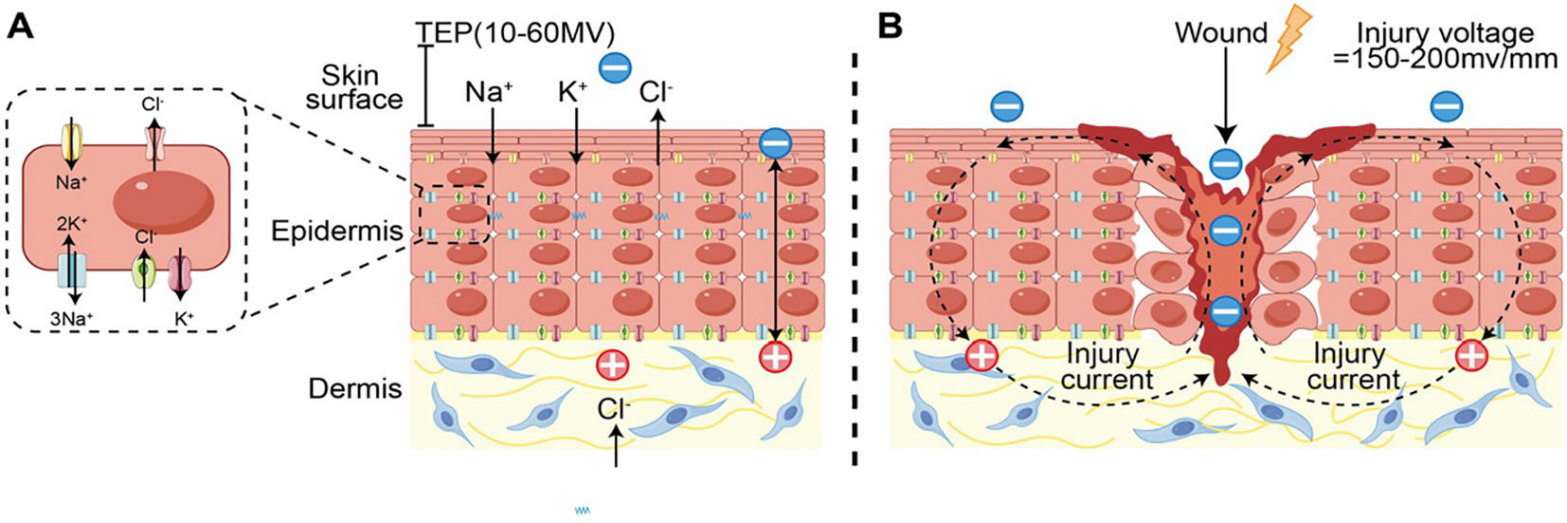
**(A)** schematic illustration of the native “skin battery” in intact epidermis. **(B)** schematic illustration of the injury current at a wound site. lonic fluxes emanating from the wound margins generate this current, and a complete electrical loop is established when the wound gap is filled with conductive fluid such as exudate or blood.

In 1983, Foulds (13) demonstrated that human skin possesses an endogenous transepithelial potential—often termed the “skin battery”—whose magnitude ranges from 10 to 60 mV, depending on the anatomical site. Two complementary factors generate this potential. First, Na^+^ and Cl^-^ channels are positioned on the apical membrane, whereas K^+^ channels and the Na^+^/K^+^-ATPase reside on the basolateral membrane. This asymmetric ion distribution drives a net inward flux of positive charge; the associated counter-movement of Cl^-^ produces a transepithelial potential difference (TEP) of 10–60 mV (14). Second, tight junctions located in the stratum granulosum 2 establish the epidermal permeability barrier and are essential for sustaining the TEP (14).Under physiological conditions the resulting electric field is oriented perpendicular to the skin surface, but it changes abruptly after injury. Disruption of the TEP allows ions to follow their concentration gradients into the wound bed, creating an electric-field gradient of roughly 150–200 mV mm^-^¹—known as the wound current or injury current, also termed the lateral current (15, 16). This current is fundamentally ionic: multiple studies show a positive correlation between the influx of Na^+^, Cl^-^, K^+^ and Ca²^+^ and the strength of the wound current (17, 18). When a conductive fluid (e.g. exudate or blood) is present, a complete electrical circuit closes. Although the influence of wound depth on current characteristics has not been fully clarified, the dermis is more conductive than the underlying fat or muscle, suggesting that the return current predominantly traverses the dermis regardless of depth (19). Throughout the different phases of wound healing, distinct cell populations exhibit directed migration into the wound area. The local electric fields generated by wound currents provide directional guidance cues for specific cell types, including keratinocytes, fibroblasts, and immune cells, thereby initiating and sustaining the healing process (see **Figure 1**). This directed migration of cells in response to an electric field is known as electrotaxis (20). Importantly, average healing rates are estimated to decline by about 25% in the absence of this current. Recognition of these bioelectrical mechanisms has catalyzed research into exogenous electrical stimulation (ES) as an adjunctive therapy to accelerate wound closure across a range of clinical settings (21).

### 2.2 Electrotaxis of cells in an electric field

At the macroscopic level, wound currents provide cells with directional cues that guide them “towards the wound”. At the microscopic level, however, the molecular mechanisms by which cells sense an electric field and convert it into signals for directed migration remain incompletely understood. Electrotaxis—directed cell migration in response to an electric field—is characterized by two key parameters: migration speed and directional bias. These two components can be regulated independently; for example, changes in directionality do not necessarily coincide with changes in speed, underscoring the complexity of the underlying mechanisms.

Babona-Pilipos et al. (22)reported that chelating intracellular Ca^2+^, lowering extracellular Ca^2+^ levels, or blocking voltage-gated calcium channels (such as L-type Cav1.2 and T-type Cav3.2/3.3) all significantly reduce migration speed, without impairing the ability of cells to orient relative to the electric field. This finding suggests the presence of parallel Ca^2+^-dependent motility machinery and Ca^2+^-independent pathways for electric field sensing.

**Figure 2:**
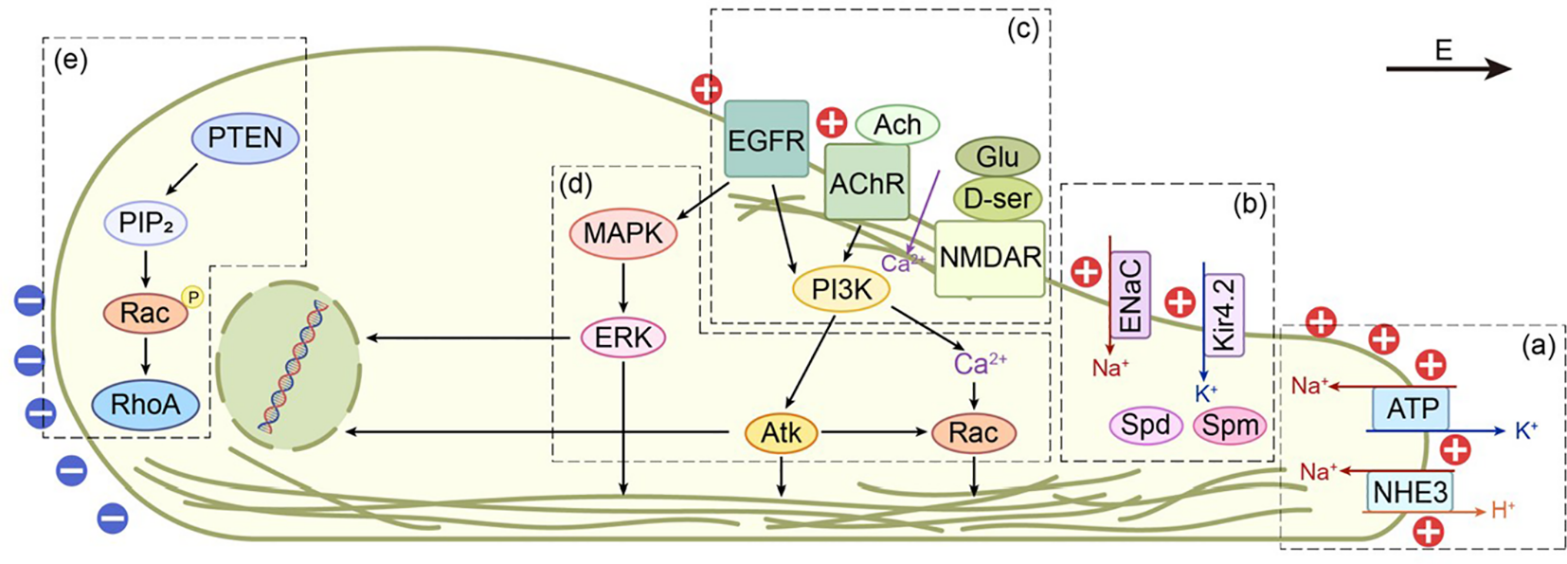
Signaling network in cells subjected to cathodal electrical stimulation. Cells polarize toward the cathode and extend a leading-edge protrusion; the front is depolarized (‘+’) whereas the rear is hyperpolarized (‘-’).**(a)** NHE3 clusters at the cathodal face and, together with the Na^+^/K^+^-ATPase, fine-tunes the membrane potential, thereby remodeling the β-actin cytoskeleton. **(b)** ENaC is likewise enriched at the cathode. The polyamines spermidine and spermine, being positively charged, accumulate at the same site and hinder outward Kir4.2 currents.**(c)** AChR and NMDAR are activated on the cathodal side of the electric field, driving Ca2^+^ influx that promotes lamellipodial extension and simultaneously activates the PI3K/Akt pathway. In contrast, EGFR is activated by the field in a ligand-independent manner, triggering both MAPK and PI3K signaling cascades. **(d)** PI3K can initiate additional responses via its downstream effector Akt or, in conjunction with PLC and Ca2^+^, activate Rac. EGFR activation further enhances the expression of MMPs, integrins, and genes related to metabolism and the cytoskeleton through the MAPK-ERK axis.**(e)** PTEN accumulates at the anodal side, elevating PIP2 levels, which leads to Rac phosphorylation anp increased RhoA expression.

Current studies indicate that the initial “sensors” for an applied electric field (EF) involve redistribution of membrane components and activation of specific ion channels. Under an EF, molecules such as EGFR (23, 24), NHE3 (25, 26), and ENaC (27) accumulate at one pole of the cell, whereas AChR (28), NMDARs (29), and Kir4.2 (30) exhibit differential patterns of activation (see **Table 1**). Inhibition of these “sensors” markedly diminishes cellular directional responses. Interestingly, Nakajima et al. (30) found that knockout of KCNJ15, which encodes Kir4.2, completely abolishes directional migration. Although Kir4.2 itself does not become polarized, PIP3 and polyamines (such as spermidine and spermine), which regulate the inward-rectifying activity of Kir channels, polarize towards the cathodal side. Downstream signaling in electrotaxis is mediated predominantly by the PI3K/Akt and MAPK–ERK 1/2 pathways. In cathode-directed cells, such as keratinocytes (31), the PI3K/Akt pathway is dominant: its activation increases PIP3 expression and Akt phosphorylation, thereby promoting asymmetric organization of the cytoskeleton. In contrast, anode-directed cells, such as macrophages (32), rely mainly on the MAPK–ERK 1/2 pathway, in which ERK phosphorylation exhibits both time- and field strength–dependent dynamics (see **Figure 2**).

**Table 1:** Relevant evidence on the mechanism of cellular galvanotaxis.

Cell type	Experimental model	ES strength and type	Manipulation or combined treatment	Sensor and signaling pathway	Reference
Adult mouse subependyma neural precursor cells (NPCs)	*In vitro* galvanotaxis (electrotaxis chamber, time-lapse microscopy)	Constant DC EF (250 mV/mm, 1.5–4 hours)	Low-Ca²^+^ media + BAPTA; Intracellular chelator (BAPTA-AM); VGCC inhibitors (Nifedipine for L-type, ML218 for T-type); RT-PCR for VGCC; F-actin immunofluorescence; Live/dead assay	L- and T-type voltage-gated calcium channels (Cav1.2, Cav3.2/3.3) are activated, leading to Ca²^+^ influx and activating the F-actin polymerization pathway (promoting protrusion extension and speed)	(22)
Human keratinocytes (HaCaT cell line)	*In vitro* electrotaxis (migration chamber)	Pulsed DC EF (50–250 mV/mm, duty cycle: 20–100%)	MEK/ERK inhibitor U0126; Parameter modulation	EGFR aggregates at the cathode side, activating the MAPK/ERK1/2 pathway	(23)
Human metastatic breast cancer cell line MDA-MB-231	*In vitro* model	Constant DC EF (1.5 V/cm, 3 V/cm; 4 V/cm, 5 V/cm)	None	EGFR aggregates at the anode with intracellular Ca²^+^ increase, activating the PI3K/Akt pathway	(24)
HEK 293 cells; Mouse corneal cells	*In vitro* electrotaxis; *In vivo* corneal wound healing	Constant DC EF (0.25–0.3 V/mm, 2 hours)	PKCη inhibitors (pseudosubstrate, edelfosine, Gö6983); NHE3 inhibitor (S3226); PKC knockout mice; Co-IP for pNHE3/PKCη/γ-tubulin; FACS for protein levels/membrane potential	Na^+^/H^+^ exchanger NHE3 is activated (phosphorylated, leading to H^+^ efflux/Na^+^ influx), activating the PKCη-dependent pathway (forming pNHE3/PKCη/γ-tubulin complex, promoting MTOC polarization and directed migration)	(25)
HEK 293 cells; Mouse corneal epithelial cells	*In vitro* electrotaxis (electrotaxis chamber, time-lapse microscopy)	Constant DC EF (0.3 V/mm, 2 hours)	NHE3 inhibitor (S3226); siRNA NHE3 knockdown; Actin inhibitor (cytochalasin D); Co-IP for pNHE3/β-actin; Immunofluorescence for patches	Na^+^/H^+^ exchanger NHE3 is activated (phosphorylated, leading to H^+^ efflux/Na^+^ influx and membrane patch formation), activating the β-actin interaction pathway (pNHE3/β-actin complex maintains directional stability)	(26)
Primary human keratinocytes (NHKs); Human keratinocytes (HaCaT)	*In vitro* galvanotaxis (electrotaxis chamber, time-lapse microscopy)	Constant DC EF (100 mV/mm)	ENaC inhibitor (amiloride), siRNA ENaC knockdown (α/β/γ subunits); Other blockers (BaCl2, TEA, niflumic acid); Na^+^-free medium; Western blot for ENaC; Fluorescence for F-actin/PIP3	Epithelial sodium channel (ENaC) is activated, leading to Na^+^ influx (asymmetric expression at the anode side), activating the PI3K signaling pathway (PIP3 polarizes to the leading edge)	(27)
Xenopus muscle cells	*In vitro* electrotaxis (custom chamber, time-lapse microscopy with QDs)	Constant DC EF (2.5 V/cm, 5 V/cm, 7.5 V/cm)	Disrupt F-actin (latrunculin A, jasplakinolide); Mutant rapsyn expression (coiled-coil deletion); Kinase-dead MuSK expression; Antibody staining for phosphotyrosine, phospho-AChR β (Y390), phospho-Src (Y418); QD tracking for trajectories	AChR is differentially activated on the cathodal side., showing EF strength dependence, activating the MuSK-Src pathway	(28)
Rat embryonic neural stem/progenitor cells (NSPCs from LGE)	*In vitro* LGE explant culture	Constant DC EF (30 50 100 250 mV/mm)	NMDAR antagonist (DAPV); Co-IP of NR2B/Tiam1/p-Pak1/actin; Immunofluorescence for NR1/NR2B; Immunoblot for p-Pak1	NMDAR is differentially activated on the cathodal side, showing EF strength dependence; PIP3 produced by PI3K recruits and activates the Tiam1-Rac1 axis	(29)
Human corneal epithelial, Human keratinocytes, (MDA-MB-231; Human glioma (U251)	*In vitro* galvanotaxis (electrotaxis chamber with PDMS stencil, time-lapse microscopy)	Constant DC EF (0–500 mV/mm, threshold ~30 mV/mm)	KCNJ15 siRNA knockdown, BaCl2 blocker; Polyamine modulation (DENSPM depletion, PUT increase); Mutant KCNJ15-E157N; Akt-PH-EGFP for PIP3; Immunostaining for polyamines/Kir4.2	Inward rectifier K^+^ channel Kir4.2 (encoded by KCNJ15) is activated, coupled with polyamines (SPM/SPD), leading to K^+^ flux changes and activating the PI3K/Akt pathway (causing PIP3 polarization)	(30)

### 2.3 Characteristics of wound current in diabetic wounds

Traditional views hold that the formation of wound currents results from the exudation of Na^+^ and K^+^ ions along their concentration gradients after the skin loses its epidermal barrier function (33). Previous studies have also reported that blocking Cl^-^ ion channels significantly reduces the wound current. However, one study (17) measured ion flows following corneal injury: 1) Ca^2+^ efflux, reaching a peak of 5 pmol/cm²/sec at 20 minutes post-injury and persisting thereafter; 2) K^+^ efflux rapidly rising to 150–200 pmol/cm²/sec, then sharply declining after 20 minutes post-injury; 3) Astonishing influx of Na^+^ at 15 nmol/cm²/sec; 4) Massive influx of Cl^-^ persisting for over 90 minutes, with a flow rate reaching 300 nmol/cm²/sec. The results indicate that the massive influx of Cl^-^ ions is the primary component constituting the wound current. Shen et al. (34) observed in wound models of different diabetic mice: 1. The current magnitude at the wound edges was significantly higher than at the center. 2. The current at the edges of diabetic wounds showed a marked decrease or even absence. 3. There is a positive correlation between wound current and wound healing.

## 3 Clinical evidence

### 3.1 Clinical evidence supporting electrical stimulation in diabetic wound healing

#### 3.1.1 Low-intensity direct current

Mohajeri Tehrani et al. (35) recruited 20 patients with Wagner grade 2 type 2 diabetes and randomized them equally to an intervention and a control group. The intervention group received electrical stimulation at 1.48 ± 0.98 mA, 1 hour per session, three times per week, for 4 weeks. The intervention group exhibited a significantly greater reduction in wound surface area (WSA) than controls. Plasma VEGF and NO levels were also markedly higher in the intervention group, suggesting that electrical stimulation may promote the release of vascular endothelial growth factor and nitric oxide, upregulate angiogenic factors, and thereby improve skin temperature and wound healing in diabetic foot ulcers (DFU).

Asadi et al. (36) enrolled 24 patients with ischemic DFU (Wagner grade 2) and found a significant increase in HIF-1α levels in wound fluid after 1 hour of electrical stimulation. As an upstream regulator of VEGF, HIF-1α modulates VEGF expression and ultimately enhances local perfusion in cutaneous wounds, indicating that electrical stimulation may improve the pro-angiogenic environment in ischemic DFU. After 4 weeks of treatment, wounds in the electrical stimulation group showed complete re-epithelialization and organized sublayers compared with controls.

#### 3.1.2 Pulsed current

Peter et al. (37) conducted a randomized double-blind trial involving 40 patients with diabetic foot ulcers. In the experimental group, participants received high-voltage pulsed current (HVPC) at 50 V and 100 pulses per second, administered five times per week for 45 min per session over 12 consecutive weeks. The mean wound area reduction in the ES group reached 86.2%, compared with 71.4% in the control group, and the proportion of completely healed wounds was 65% in the ES group versus 35% in the control group.

Baker et al. (38) evaluated both symmetric biphasic rectangular pulsed currents and asymmetric biphasic rectangular pulsed currents in patients with diabetic foot ulcers. They found that treatment with asymmetric biphasic pulsed current more than doubled the wound healing rate, whereas the healing rate achieved with symmetric biphasic pulsed current did not differ significantly from that of the control group.

Zulbaran-Rojas et al. (39) evaluated a home-use device (E-Stim) delivering high-voltage sinusoidal pulses at 150–250 V. Patients self-administered 1 hour daily for 4 weeks, resulting in significantly higher tissue oxygen saturation at the wound site compared with controls.

Tian et al. (40) treated 27 cases of diabetic ulcers using pulsed current at 100 Hz and 15 mA for 30 min once daily. Compared with controls, the intervention group showed marked improvements in wound healing, infection control, and local blood-flow perfusion.

**Figure 3:**
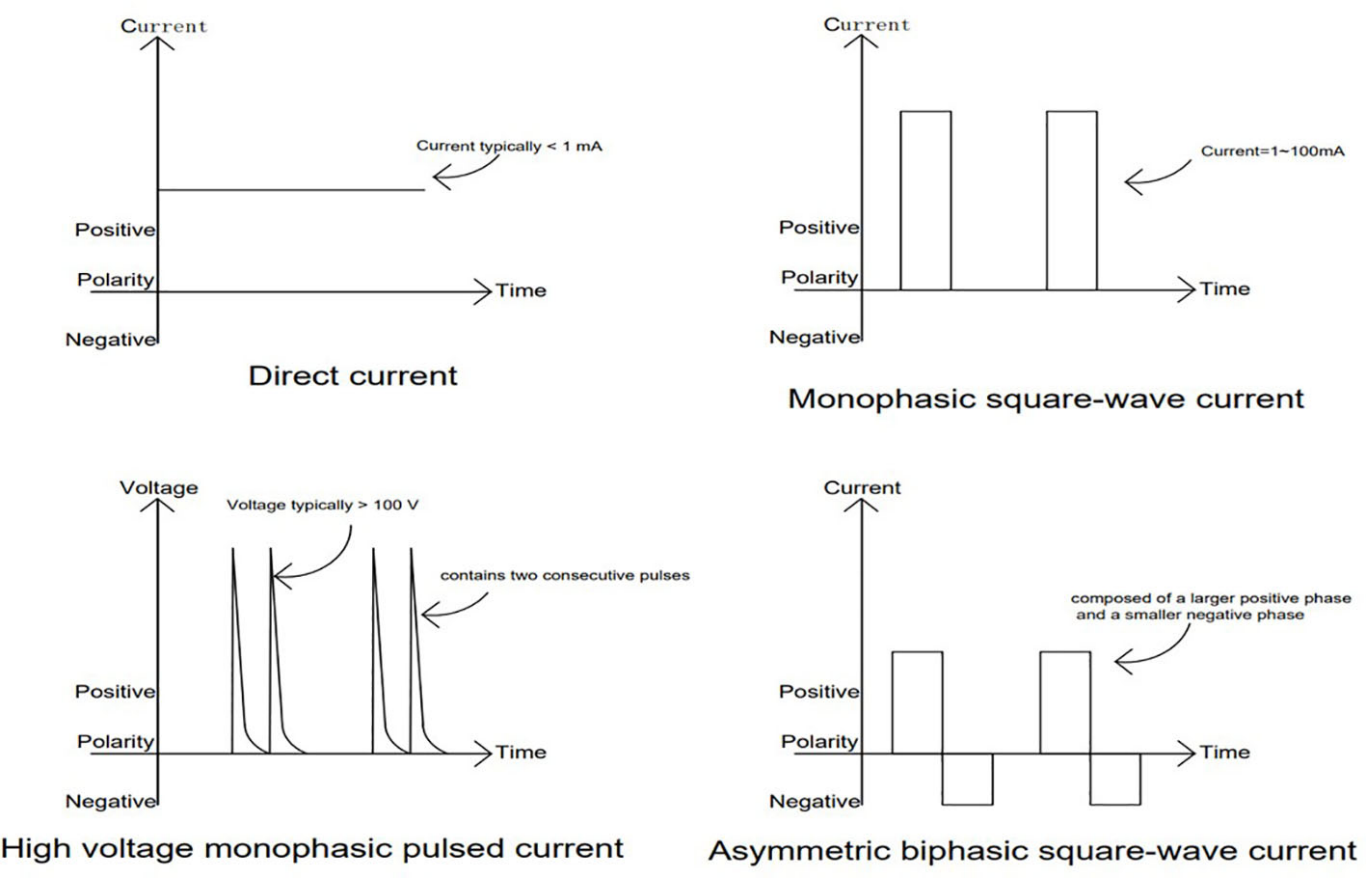
Four common electrical-stimulation waveforms: direct current (DC), monophasic square-wave pulses, high-voltage monophasic pulses, and asymmetric biphasic square-wave pulses.

Burdge et al. (41) retrospectively assessed 30 DFU patients who received adjunctive HVPC after failing prior multidisciplinary wound care. With standard debridement, infection control, and glycemic management maintained, 8 weeks of HVPC yielded a 55% complete healing rate, an average 75% reduction in wound area, and a 40% improvement in perfusion. Characteristics of the different currents are shown in **Figure 3**.

#### 3.1.3 Combined therapies

Petrofsky et al. (42) conducted a randomized controlled trial in patients with diabetic foot ulcers, combining biphasic electrical stimulation (ES; 20 mA, 30 Hz; 30 min/session, 3 sessions/week, for 4 weeks with intermittent scheduling) with red-light therapy. Compared with red-light therapy alone, the combination produced greater improvements in local blood flow and larger reductions in wound surface area. The synergy between ES and red light may stem from a shared mechanism—namely, increased release of the vasodilator nitric oxide (NO), which augments perfusion.

### 3.2 Current limitations of clinical studies

Although multiple clinical studies suggest that electrical stimulation (ES) may confer potential benefits as an adjunctive therapy for diabetic foot ulcers (DFUs) (see **Table 2**), the overall methodological quality of the available evidence remains limited (see **Table 3**).

**Table 2:** Summary of evidence for electrical stimulation therapy in diabetic wound healing: diabetic foot ulcer patients, animal models, and cellular studies.

Author	ES modality	Model	DFU subtype	Ulcer grade	Device parameters	Treatment course	Electrode placement and distance	Combined treatment	Adverse events	Efficacy indicators
Mohajeri-Tehrani et al. (35)	LIDC	Human: 10/10	Neuropathic	Wagner 2	BTL-5000 1.48 ± 0.98 mA	3 d/wk,1 h/d, for 4 weeks	Active: lateral margin of the ulcer; Passive: 20 cm proximal to the active electrode	Standard treatment	Not reported	Biomarkers: VEGF increased significantly in the ES group; NO increased significantly after the 12th session; and the wound surface area (WSA) in the ES group was reduced by more than 31% after 12 sessions, skin temperature significantly increased
Asadi et al. (36)	LIDC	Human: 15/15	Ischemic + Neuropathic	Wagner 2	BTL-5000 3.36 ± 0.58 mA	3 d/wk,1 h/day, for 4 weeks	Active: proximal edge of the ulcer; Passive: 20 cm proximal to the active electrode	Standard treatment	Not reported	Biomarkers: HIF-1α significantly increased in wound fluid. Area reduction rate: WSA reduction of 59.49% after 12th session in ES group (*vs* control 27.07%),
Peter et al. (37)	HVPC	Human: 21/19	Not reported	Texas 1A-2A	Micro-Z™ 50V, 80pps, 0.8%	5 d/wk,45 min/d, for 12 weeks	Active: center of the ulcer; Passive: 15–20 cm proximal to the wound	Standard treatment	Not reported	ES group average reduction 80% (*vs* placebo 44%). Complete closure: 60% in ES group *vs* 29% in placebo. Healing time: Faster in ES group
Baker et al. (38)	Biphasic square-wave pulse	Human: 61/53	Not reported	Not reported	UltraStim A: 50 pps,0.5% B: 50 pps,1.5%	1 time/day, 1.5h/d, for 4week	Active: proximal to the ulcer; Passive: distal to the ulcer	Standard treatment	Not reported	Higher cure rate in the asymmetric biphasic square−wave group *vs*. control
Zulbaran-Rojas et al. (39)	HVPC	Human: 16/17	Not reported	Not reported	Tennant Biomodulator^®^ PRO 150–250 V	1h/d, for 4 weeks	Active: above the ankle joint; Passive: not reported	Standard treatment	Not reported	4-week area reduction rate: 22%. Complete closure: 2/16 complete (2 cases in IG healed early). Blood perfusion: SatO2 improved; Ulcer depth reduction (IG: 23.5% *vs* CG 0.8%); VPT improved (at 2W, IG better than CG)
Tian et al. (40)	LFES	Human: 25/25	Not reported	Not reported	Myomed632VUX15mA, 100pps	5 times/week, 0.5h/d, total 4–8 weeks	Active: lateral margin of the ulcer; Passive electrode: lateral margin of the ulcer	Standard treatment	Occasional minor tingling, well-tolerated, no skin damage	Higher cure rate in the ES group *vs*. control blood perfusion improved in the ES group *vs*. control
Burdge et al. (41)	HVPC	Human: 20	Not reported	Not reported	Intelect Legend XT 120 V 100 pps, 1%	3-5d/wk,1h/d, 8 weeks	1. Wound site; 2. Ankle joint; 3. Gastrocnemius muscle; 4. Thigh	Standard treatment	occasional minor skin erythema, no withdrawals or complications	Area reduction rate: Average 75%. Complete closure: 55% (11/20). Average healing time: 8 weeks. Blood perfusion: Significant improvement (laser Doppler +40%)
PETROFSKY et al. (42)	Symmetry biphasic sine-wave pulse	Human: 10/10	Not reported	Not reported	Challenge 8000 A,20 mA, 30 pps	3d/wk,0.5h/d, for 4 weeks	Active: lateral margin of the ulcer; Passive electrode: Not reported	Standard treatment + red light	Not reported	Area reduction rate: Local red light + ES group 60%; Control 20%. Blood perfusion: Significant increase in local red light + ES group (laser Doppler +30-40%)
Wang et al. (44)	HVMPC, LVMPC, DC, HVMPC+DC, HVBPC, LVBPC	Diabetic rats, Human umbilical vein endothelial cells (HUVEC)	N/A	N/A	Flexible ES device; HVMPC 40 V, LVMPC 4 V, HVBPC 40 V, LVBPC 4 V; (all: 100 pps, 1%) DC 4 V;	Every 2 days 1h, 1h/d, for 19 days	Active: inserted into wound and on wound surface; Passive: not reported	Standard treatment + CVG dressing	Inserted electrode: Burns (most severe in DCHVMPC group, followed by high-voltage group).	Biomarkers: VEGF↑, p-Akt↑, p-ERK1/2↑; Smad2/3↓. Area reduction rate: HVMPC+CVG group 85-90% (19 days, *vs* control 50-60%). Complete closure: Faster in HVMPC+CVG (~19 days, *vs* control >25 days). Blood perfusion: Not directly measured, but VEGF/CD31↑ suggests improvement. Other: Accelerated epithelialization, reduced scarring (area/width decreased); HUVEC proliferation/migration↑ (EdU+ cells↑, migration rate↑)
Wang et al. (50)	Piezoelectric self-powered microcurrent	Diabetic rats, L929 fibroblasts,HUVEC	N/A	N/A	PVA/PVDF piezoelectric hydrogel dressing	Related to animal movement, 14 days	Hydrogel directly covers the wound	None (hydrogel only)	Not reported	Biomarkers: VEGF↑, p-Akt↑, p-ERK1/2↑; Col I/III↑; Inflammatory factors↓ (IL-6/IL-1β/TNF-α↓). Area reduction rate: PVA/PVDF group 90% (14 days, *vs* control 50%). Complete closure: Faster. Blood perfusion: Improved (CD31/α-SMA↑, vascular density↑). Other: Collagen deposition↑; Accelerated re-epithelialization; (EGF/FGF/VEGF) ↑
Abedin-Do et al. (51)	LIDC	Fibroblasts extracted from skin of diabetic patients	N/A	N/A	Custom conductive PPy/HE/PLLA membrane 20, 40, 80, 100 mV/mm	6 or 24 h ES, then 48 h culture	N/A	None	High intensity (80–100 mV/mm) caused cytotoxicity (LDH↑);	Biomarkers: IL-6/IL-8↓; GM-CSF/IL-1β/FGF7↑. Other: Promoted cell survival and growth (survival rate↑, cell count↑ ~1.2-1.4 times); ES effects maintained up to 5 days later (20, 40 mV/mm groups)

Device Parameters, This column lists the name of the device along with its operational parameters. For example, “Intelect Legend XT 120 V 100 pps, 1%” specifies the following; Device Name, Intelect Legend XT; Voltage, 120 V; Frequency, 100 Pulses Per Second (pps); Duty Cycle, 1%; N/A means Not Applicable. Standard treatment including debridement, wound cleaning with saline, dressing, and systemic antibiotic therapy.

**Table 3:** Limitations of clinical trials of ES treatment for DFUs.

Study	Study design	Main limitations	Specific issues/reasons
Mohajeri-Tehrani et al. (35)	RCT	Small sample size; large inter-individual variability in current intensity; lack of clearly defined endpoint outcomes	N=20; current intensity 1.48 ± 0.98 mA; only % wound area reduction reported, without time to healing or healing rate
Asadi et al. (36)	RCT	No stratification by DFU subtype; large inter-individual variability in current intensity; no clearly defined endpoint outcomes.	Included both neuropathic and ischemic DFUs without stratified analysis; current intensity 3.36 ± 0.58 mA; only % wound area reduction reported, without time to healing or healing rate.
Peter et al. (37)	RCT	Different DFU grading system; use of a self-designed device, limiting generalizability	Used the Texas classification system and a self-designed Dacron-mesh silver nylon stocking as the intervention device
Baker et al. (38)	RCT	Heterogeneous population; no DFU type or grading; lack of blinding.	Included both outpatients and inpatients; DFU type/grade not reported; no double-blinding, and some patients were crossed over between groups
Zulbaran-Rojas et al. (39)	RCT	Incomplete ES parameters; no DFU type or grading; data quality concerns with home-based self-administration.	Only voltage reported (no frequency or duty cycle); DFU type/grade not described; ES delivered at home with questionnaire-based outcome reporting.
Tian et al. (40)	RCT	Incomplete ES parameters; no DFU type or grading; lack of blinding.	Voltage details not reported; DFU type/grade not described; no blinding procedures
Burdge et al. (41)	Retrospective study	Retrospective design; highly heterogeneous treatment protocol	Only refractory DFUs included without grading; wide variation in treatment frequency; four electrode configurations, mixing local wound ES and neuromuscular stimulation
Petrofsky et al. (42)	RCT	Small sample size; combined therapy; unclear electrode placement; no DFU type or grading; lack of blinding; no clearly defined endpoint outcomes.	N=20, limiting interpretation of ES-specific effects; placement of the passive electrode was not clearly described; no blinding; key endpoints such as time to healing and healing rate were not reported

First, most trials are single-center studies with small sample sizes (in some cases including only approximately 20–40 participants), resulting in clearly insufficient statistical power. Second, there is substantial heterogeneity in the ES protocols applied across studies: some used low-intensity direct current, whereas others employed pulsed currents; some reported voltage as the primary dosing parameter, while others focused on current intensity. In addition, several key parameters—such as stimulation frequency and duty cycle in the studies by Baker and by Zulbaran Rojas—were not reported at all, making direct comparison between studies virtually impossible. Third, many trials provide inadequate information on blinding procedures, and some investigations are even retrospective observational studies, which further increases the risk of bias. Moreover, although ulcer grading is reported in most studies, the phenotypic characterization of patients (e.g., ischemic *vs*. neuropathic *vs*. mixed DFUs) is generally insufficient. Electrode placement and configuration also vary widely between studies and remain a matter of debate, adding another layer of heterogeneity to the evidence base. With respect to outcomes, most trials focus on surrogate endpoints such as reduction in wound area, local perfusion, or biomarker levels, whereas key clinical outcomes—including time to complete healing, recurrence rate, amputation rate, health-related quality of life, and systematic reporting of adverse events—are comparatively underreported.

Taken together, these limitations substantially weaken the strength of evidence supporting ES for DFUs and underscore the urgent need for large-scale, multicenter randomized controlled trials with standardized ES parameters and rigorously defined, clinically meaningful outcome measures.

### 3.3 Safety considerations

When applying electrical stimulation (ES) for the treatment of diabetic foot ulcers (DFUs) in clinical practice, ES should be used strictly within clearly defined indications and safety boundaries, and always on the basis of standard wound care and optimal systemic management. Synthesizing current guidelines on ES and electro-physical therapies, device instructions, and evidence from systematic reviews on DFUs, the following situations are generally regarded as relative or absolute contraindications.

Patients with implanted cardiac pacemakers or implantable cardioverter-defibrillators (ICDs), in whom the electrodes or current pathway may traverse or approach the precordial region, are at potential risk of interference with the cardiac conduction system. In the presence of uncontrolled severe local or systemic infection (e.g., cellulitis, sepsis), ES cannot substitute for adequate antimicrobial therapy and surgical debridement. Moreover, several randomized controlled trials (RCTs) of ES in DFUs explicitly excluded infected ulcers at enrolment (35, 36, 43), indicating that both safety and efficacy data are lacking in this population. Where ulcers are associated with exposed bone, tendon, or major blood vessels, local tissue tolerance is reduced and current distribution is difficult to predict, which theoretically increases the risk of thermal injury and deep tissue damage. In patients with suspected malignant ulcers or tumor involvement at the wound margin, there is currently no robust evidence regarding the safety of ES in malignant lesions, and many commercial devices list “known or suspected local malignancy” as a formal contraindication in their instructions for use; therefore, routine ES is generally not recommended until malignancy has been adequately excluded. Similarly, in patients with severe, unreconstructed lower-limb ischemia and markedly compromised perfusion who are not candidates for revascularization, most DFU RCTs have used “irreversible severe ischemia” as an exclusion criterion (35, 36, 41), suggesting that both the effectiveness and safety of ES in this subgroup remain uncertain.

Available animal and clinical data indicate that ES-related adverse effects are mainly limited to local thermal injury, pH alterations, and skin irritation (41). In animal experiments, the direct application of relatively high-voltage direct current to the wound surface has been shown to induce local thermal damage (44). This finding highlights the need for careful control of current density and the duration of individual stimulation sessions, particularly when wound resistance is high or invasive electrodes are used. In routine clinical practice, non-invasive surface electrodes are therefore preferable, combined with a “low-intensity initiation and gradual escalation” strategy. Prolonged continuous exposure to direct current, even at low current intensities, can induce alkalinization of tissues beneath the anode and acidification beneath the cathode (45), potentially creating a suboptimal microenvironment for cellular processes. By contrast, low–duty cycle pulsed currents substantially reduce the average exposure time of cells to the electric field and are considered relatively safer in this regard (46).

In clinical RCTs and systematic reviews, most reported adverse events are mild cutaneous reactions, such as erythema, pruritus, or eczema-like changes (47, 48). These events are usually attributable to the electrode or dressing materials and the conductivity medium, and in the vast majority of cases can be alleviated by changing materials, reducing wear time, and optimizing local skin care. Severe adverse events are extremely rare and are generally underreported (43, 49). Nevertheless, it is important to recognize that patients with DFUs are typically elderly and burdened with multiple chronic comorbidities. ES protocols often require daily or alternate-day sessions lasting 30–60 minutes, and long-term adherence is inherently challenging. Systematic reviews have consistently identified small sample sizes, short follow-up durations, and insufficient monitoring of adherence as common limitations of existing studies (49).

With the emergence of wearable and home-based ES devices (39), treatment accessibility has improved to some extent. However, studies also indicate that home-based use introduces new safety challenges, including inaccurate electrode placement, non-standard parameter settings, delayed recognition of wound deterioration, and lack of structured remote monitoring. These issues need to be carefully addressed in future device design, clinical protocols, and follow-up strategies to ensure that expanded access does not come at the expense of patient safety.

## 4 Preclinical evidence

Wang et al. (44) Compared multiple electrical stimulation paradigms—high-voltage monophasic pulsed current (HVMPC), low-voltage monophasic pulsed current (LVMPC), direct current (DC), HVMPC+DC, high-voltage biphasic pulsed current (HVBPC), and low-voltage biphasic pulsed current (LVBPC). HVMPC at 40 V, 100 pulses per second (pps), and 1% duty cycle produced markedly greater wound surface area reduction beginning on day 7 versus all comparators. The HVMPC group exhibited faster re-epithelialization, reduced inflammatory cell infiltration, and more mature granulation tissue. Collagen deposition increased with more ordered fiber alignment; scar area and width were significantly reduced (p<0.05). VEGF and CD31 were significantly upregulated, while Smad2/3 was downregulated. Concomitantly, p-Akt and p-ERK1/2 levels were significantly elevated (p<0.05), indicating activation of PI3K/Akt and ERK1/2 pathways that promote cell proliferation and migration. Adding a flexible adjunctive dressing (Chitosan-Vaseline gauze) to HVMPC yielded the most optimal healing outcomes. Importantly, early experiments that placed electrodes within the wound cavity caused burns under high-voltage pulses (40 V) and DC (4 V). After switching to surface-placed electrodes, no burns or other adverse events were observed.

Wang et al. (50) designed a piezoelectric nanogenerator composed of a self-powered PVA/PVDF composite hydrogel. Under external force, the internal dipole moments of this piezoelectric material rearrange, causing charges to accumulate on different crystal faces, manifesting as voltage/charge at both ends. In diabetic rats, a 1 cm diameter full-thickness skin defect was created on the back. In the electrical stimulation group, the wound completely closed by day 10 (compared to over 14 days in the control group), with a closure rate increased by 20-30%. The electrical stimulation group exhibited enhanced re-epithelialization (increased epidermal thickness), collagen deposition (1.5-fold increase in collagen content), and angiogenesis (2-fold increase in neovascular density). It promoted the polarization of macrophages from pro-inflammatory M1 to anti-inflammatory M2 phenotype (increased expression of M2 markers) and reduced levels of inflammatory factors (IL-6/IL-1β/TNF-α) by 40-60%. The researchers also designed *in vitro* scratch migration assays and Transwell migration assays, finding that the cell migration speed in the electrical stimulation group was significantly superior to that in the control group.

Abedin-Do et al. (51) Exposed diabetic human dermal fibroblasts to direct-current (DC) electric fields of 20, 40, 80, and 100 mV/mm. Low-to-moderate field strengths (20–40 mV/mm) significantly enhanced cell adhesion and proliferation, reduced pro-inflammatory cytokine secretion (e.g., IL-6, IL-8), and increased FGF-7 production. In contrast, higher intensities (80–100 mV/mm) adversely affected cells, reducing viability; such high fields are therefore not recommended for clinical translation.

## 5 Emerging technologies

### 5.1 Incorporation of novel conductive materials

Electrical stimulation therapy relies on the careful development and application of novel materials with unique physicochemical properties, designed to establish ideal interactions with biological tissues (52). Incorporating electroactive conductive polymers into polymeric biomaterials is a fundamental step in fabricating conductive wound dressings. Integrating conductive polymers (CP) into wound dressings can enhance antimicrobial activity, promote cell proliferation, and enable controlled drug release or electrical stimulation through the application of external currents (53). To date, numerous conductive wound dressings have been designed in various forms, such as films, membranes, hydrogels, and electrospun dressings (54). The conductive composite membrane developed by Shi and colleagues was formed by blending polypyrrole (PPy) with polylactic acid (PLLA) (55). Upon electrical stimulation, fibroblasts on this conductive composite membrane exhibited better adhesion and proliferation compared to those on tissue culture polystyrene dishes. Later, they found that the ES-mediated conductive PPy/PLLA membrane (100 mV/mm) extended the survival time of human skin fibroblasts by regulating cytokines (e.g., IL-6 and IL-8) (56). Hydrogels, as an emerging material for diabetic foot treatment, incorporate naturally derived components (such as alginate, collagen, etc.), conferring excellent biocompatibility and antimicrobial properties (57, 58). Combining electrical stimulation with conductive hydrogels (e.g., hyaluronic acid-chitosan/graphene oxide) not only promotes cell migration but also exhibits enhanced antimicrobial characteristics (59).

### 5.2 Novel power supply systems

Han et al. (60) utilized a self-powered patch (e.g., flexible photovoltaic electrodes) capable of converting light energy into electrical energy, providing sustained power for electrical stimulation while exhibiting excellent flexibility and biocompatibility. Negative pressure wound therapy (NPWT) is a common clinical technique for treating chronic wounds. Luo et al. (61) found that NPWT leads to the loss of wound electrolytes (Na^+^, Cl^-^, K^+^, etc.), thereby weakening the wound current. They combined NPWT with electrical stimulation by embedding a triboelectric nanogenerator (TENG) into the negative pressure suction sponge, where the negative pressure provides the power source, and the periodic deformation of the dressing drives contact-separation in the TENG friction layers to generate electron flow and form an electric potential, enabling simultaneous negative pressure suction and electrical stimulation of the wound.

### 5.3 Combination therapies

Studies have compared microcurrent stimulation therapy and NPWT in treating burn wounds, finding that microcurrent stimulation effectively reduces wound size but may be slightly less effective than NPWT in reducing total bacterial counts (62). Lang et al. (63) employed customized hollow carbon fiber ring electrodes and needle-like flexible electrodes. Placing the electrodes under a negative pressure drainage device, keratinocytes displayed regular shapes, uniform alignment, and movement toward the wound center, forming neat squares. Wang et al. (64) designed a stretchable, wirelessly powered photoelectric synergistic patch that achieves a coordinated effect where the electric field first directs cells to “line up,” followed by light accelerating proliferation and neovascularization, significantly enhancing skin wound repair efficiency. Additionally, due to advancements in tissue engineering and regenerative medicine, biomaterials derived from mammalian extracellular matrix (ECM) can provide mechanical support and biochemical signals for tissue regeneration, often loaded with bioactive cells or molecules for more severe wounds, such as open and chronic wounds with poor regenerative capacity (65). So et al. (66) discovered that combining electrical stimulation with ECM biomaterials enhances wound healing speed, with the rate influenced by the waveform. Electrical stimulation can also participate in drug delivery at the wound site. Soluble polymer microneedle (MN) patches have attracted significant research interest as drug delivery systems. MN patches have been used for transdermal delivery of various substances, including small-molecule drugs, proteins, and cytokines (67). Yang et al. (68) utilized a microneedle-based self-powered transdermal electrical stimulation system (mn-STESS), which converts mechanical energy into electrical energy via a sliding triboelectric nanogenerator (TENG) to provide microcurrents (approximately 1 μA) for electrical stimulation. The microneedles penetrate the skin, conducting current into the skin while continuously releasing the growth factor EGF and performing electrical stimulation. Electrical stimulation may enhance cell membrane potential differences and ion permeability, increasing cellular sensitivity to growth factors and thereby promoting cell proliferation and migration. Simultaneously, self-powered electrical stimulation enhances EGF drug efficacy, demonstrating potential to address EGF pharmacokinetic challenges.

### 5.4 Portability and programmability

Kim et al. (69) designed epidermal electronic systems (EES) that match the thickness, elastic modulus, and bending stiffness of skin, enabling seamless adhesion to the skin surface. Unlike traditional electronic devices, EES attach via van der Waals forces, achieving lightweight and flexible designs. Power supply is realized through miniature solar cells and wireless charging technology, enhancing portability. Future ES systems can be programmed to deliver specific electrical patterns tailored to different stages of wound healing or to respond in real-time to changes in the wound environment, optimizing treatment outcomes for various wound types (70). Jiang et al. (71) designed a wireless closed-loop smart bandage integrating sensors and stimulators, consisting of wirelessly powered sensing and stimulation circuits with tough hydrogel electrodes made from biocompatible conductive polymers at the tissue interface. The smart bandage hardware embeds multiple sensors to detect conductivity, temperature, pH, etc., and provides electrical stimulation in response to changes in the wound environment. Future efforts should focus on developing miniaturized, wearable, or non-invasive patch-type devices, integrating features such as automatic adjustment of treatment parameters, seamless data uploading, and remote medical monitoring to improve patient compliance and physician management efficiency (72).

## 6 Future directions

### 6.1 Current barriers to clinical translation

From fundamental mechanistic studies to early clinical trials, electrical stimulation (ES) has shown promising potential for promoting the healing of diabetic foot ulcers (DFUs). However, its widespread adoption in routine clinical practice is still hindered by several important barriers, which can be summarized as follows:

(1) Limitations in Evidence Quality and Guideline Development.

Most existing randomized controlled trials (RCTs) are single-center studies with small sample sizes and substantial risk of bias. Stimulation protocols and outcome measures vary widely across studies, and essential parameters—such as frequency, duty cycle, electrode placement, and polarity—are often incompletely reported. This results in marked heterogeneity, making it difficult for systematic reviews and meta-analyses to define clear dose–response relationships or to identify optimal treatment regimens. Consequently, clinical practice guidelines are unable to provide concrete and operational recommendations.

(2) Barriers Related to Treatment Protocols and Device Design.

ES therapy involves multiple adjustable dimensions, including current/voltage, waveform, frequency, duty cycle, electrode polarity and placement, and overall treatment schedule. Device settings vary considerably among manufacturers, and there is no standardized terminology or labeling system. Many commercial units still depend on external power supplies and multiple lead wires, making them bulky and inconvenient to wear. As a result, they are difficult to integrate seamlessly into routine wound care procedures (e.g., dressing changes, negative-pressure therapy) and are less suitable for use in primary-care settings or at home.

(3) Safety Concerns and Regulatory Challenges.

As discussed earlier, robust safety and efficacy data are lacking for high-risk populations, such as patients with implanted cardiac pacemakers, uncontrolled severe infections, ulcers with exposed bone, or suspected malignant lesions. In real-world practice, clinicians therefore tend to be cautious or even reluctant to use ES in these groups. In addition, home-use and wearable ES devices must comply with multiple layers of regulation encompassing both medical device approval and electrical safety, including electromagnetic compatibility, long-term wear safety, and capabilities for remote monitoring. These regulatory hurdles inevitably slow the translation of ES technologies from the laboratory to the marketplace.

### 6.2 Selection and standardization of electrical stimulation modes, parameters, and treatment duration

Electrical stimulation therapy has been applied clinically in patients with diabetic foot ulcers, demonstrating advantages in promoting wound healing, enhancing local blood supply to the wound, improving wound hypoxia, and reducing inflammatory responses. Low-intensity direct and pulsed currents have been used in clinical settings, while nanogenerators remain in the preclinical stage. Comparing the characteristics of these three types of electrical stimulation will further facilitate future clinical work in diabetes treatment. Nanogenerators are devices that convert mechanical energy into electrical energy, addressing issues in wound treatment such as patients being tethered by wires and discomfort in wearing, thereby improving patient compliance and aligning with the future trend of home-based treatment for chronic wound repair. Long et al. (73) demonstrated an electrical wound-healing bandage based on a wearable self-powered triboelectric nanogenerator (TENG). The device is flexible like a bandage (based on PET, Cu foil, and PTFE materials, with a thin total thickness and low bending modulus of only 0.5 GPa), making it easy to wrap around the body. Nanogenerators made from conductive hydrogels also serve dual roles in absorbing wound exudate and maintaining wound moisture. However, the energy from nanogenerators relies on mechanical motion, and the irregularity of biological movements results in uneven energy output, which is even difficult to monitor. Moreover, wound repair depends on local immobilization, which contradicts the fundamental principle of nanogenerators. Even though Luo et al. (61) used pressure changes from negative pressure devices to replace mechanical motion, the uneven pressure from negative pressure devices may adversely affect wound healing. Low-intensity direct current is a form of exogenous stimulation that closely resembles the wound currents to tissues. However, due to its prolonged duration and monotonic polarity, it can easily cause local thermal damage (as seen in Wang et al. (44), who reported tissue burns with 4V DC) and alter the local pH levels (for instance, direct currents with prolonged intensities below 1 mA can also lead to changes in local acidity and alkalinity). In contrast, pulsed current, due to its low duty cycle [with duty cycles close to 1% used by Peter et al. (37) and Burdge et al. (41)], can effectively reduce the occurrence of adverse events. Moreover, by maintaining a smaller duty cycle, it permits a larger range of selectable voltage amplitudes. Therefore, from the perspective of balancing safety and efficacy, high-voltage but low-duty cycle pulsed currents may represent a more viable option at present.

In pulsed currents, key parameters include voltage, frequency, and duty cycle. Experimental studies have shown that cell electrotaxis is closely related to voltage intensity and duty cycle, but less so to frequency (31). Pulsed currents can be categorized into high-voltage and low-voltage pulses based on intensity; they can also be divided into monophasic and biphasic pulses based on their phase characteristics. Low-voltage pulsed currents (usually with voltages <10 V) have relatively poor penetration, and some studies have found that their effectiveness in wound healing is not ideal (44). Biphasic symmetric pulse currents, due to their overall charge balance and lack of fixed polarity, may weaken the directional guidance provided by electrical stimulation. There are significant differences in cellular electrotactic responses among various waveforms of pulsed currents (46), but systematic comparative studies of different waveforms are still lacking. Currently, rectangular pulse waveforms are the most widely used in electrical stimulation therapy for wounds. Based on existing clinical and experimental data, high-voltage, low-duty cycle monophasic rectangular pulse currents may represent a relatively superior electrical stimulation approach in clinical settings, while this evaluation still needs further validation in prospective randomized controlled trials.

Regarding treatment duration, most clinical studies targeting diabetic foot ulcers adopt a treatment frequency of once daily or every other day, with single treatment durations typically ranging from 30 to 60 minutes and total treatment durations varying from 4 to 12 weeks. Some studies tend to deliver higher frequency stimulation during the early inflammatory phase of the wound to quickly restore the injury current and improve local microcirculation; thereafter, they gradually adjust the treatment frequency and overall duration based on granulation growth and wound size reduction. Overall, there is considerable variability among studies concerning the starting time point, single treatment duration, and total treatment duration, making it currently impossible to derive a “unified optimal” treatment regimen. Future multicenter randomized controlled studies should systematically compare different combinations of frequency, single treatment duration, and total treatment duration through stratified designs to establish more practical recommendations for treatment schedules.

### 6.3 Electrode placement and polarity strategies: from experience to evidence-based practices

**Figure 4:**
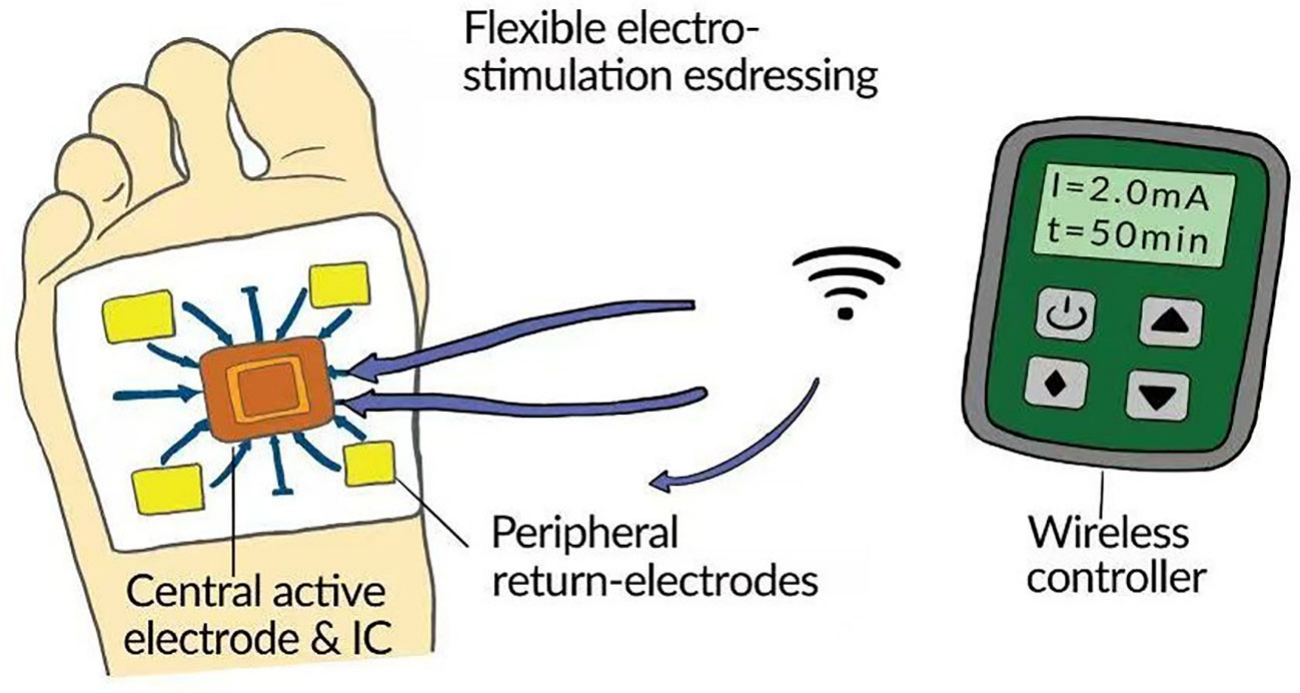
A remotely controllable advanced dressing comprising a central section anda peripheral section. The central section houses an active electrode and an on-board control unit capable of receiving external commands to deliver programmable electrical stimulation. The peripheral section contains four reference electrodes that, together with the active electrode, complete the current loop and generate a uniformly distributed directional electric cue across the wound bed.

Clinical practice predominantly employs portable transcutaneous electrical-stimulation (TES) devices for treating diabetic wounds. A typical TES unit comprises a central controller linked to several electrodes, although some investigators have adopted medium-sized systems such as the BTL-5000 that integrate ES with adjunct modalities, notably therapeutic ultrasound. During application, the active electrode is placed on moistened gauze covering the wound, whereas dispersive electrodes are affixed to intact skin located at least 15 cm—and in some protocols ≥ 20 cm—from the active site. In conventional TENS therapy, electrode placement directly on the wound is contraindicated, likely because the stimulation intensities used in TENS (often ≤ 30 mA) far exceed those deemed safe for wound ES (< 5 mA). Whether maintaining a dispersive-electrode distance of ≥ 15 cm is biologically justified remains contentious. The 2019 European guideline on pressure-injury management endorses ES (74), and most clinical studies similarly position the dispersive electrode ≥ 15 cm from the reference electrode. Conversely, several authors argue that the therapeutic field generated by wound ES is highly localized (75); excessive separation may therefore limit current delivery to peri-wound cells. Indeed, many foundational experiments that shaped current concepts in wound ES placed the dispersive electrode immediately adjacent to the wound edge, yet only a few clinical trials have replicated this arrangement. In routine care, for example, when a diabetic-foot ulcer is situated on the dorsum of the foot, clinicians often attach the dispersive electrode to the proximal fibula, inadvertently stimulating lower-leg nerves such as the common peroneal. Such configurations may thus involve mechanisms beyond simple enhancement of endogenous wound current. Burdge et al. (41), for instance, positioned four electrode pads on the wound bed, ankle, gastrocnemius, and thigh, delivering neuromuscular ES simultaneously with wound ES. Furthermore, placing a single dispersive electrode on one side of the wound may activate peri-wound cells unilaterally; increasing the number of dispersive electrodes (see **Figure 4**) or reshaping them into a circumferential (ring) design could distribute directional electric cues more uniformly around the wound.

The polarity settings of the treatment electrodes are equally crucial. Among the cells involved in wound healing, macrophages and neutrophils tend to migrate towards the anode, while fibroblasts, keratinocytes, mesenchymal stem cells (76), and endothelial cells typically show a preference for migrating toward the cathode. Therefore, if the treatment goal is to enhance inflammation clearance and antibacterial effects, it may be advisable to place the anode at or near the wound site; conversely, to promote granulation and epithelialization, the cathode is more suitably positioned at the wound or its margins. In clinical studies on diabetic foot ulcers, most protocols opt for a treatment frequency of once daily for one hour or every other day, primarily featuring continuous cathodal stimulation, where the treatment electrode serves as the cathode throughout the treatment duration.

Some early studies on other types of chronic wounds have employed alternating cathodal and anodic strategies (e.g., 3 days of cathodal stimulation followed by 3 days of anodic stimulation or 1 week of cathodal followed by 5 weeks of anodic stimulation), theoretically aiming to balance the anti-inflammatory effects of the anode with the proliferative effects of the cathode. Results from Polak et al. (77) indicated that the electrode alternation strategy did not demonstrate any additional advantages over simple cathodal stimulation within a 6-week period. On the contrary, their estimates suggested that with continuous use of the cathode, the average time to achieve a 50% reduction in wound surface area (WSA) was approximately 1.92 weeks, while the combined cathode-anode approach required about 2.6 weeks to reach the same outcome. Although the optimal polarity settings still need further validation through clinical studies, existing evidence supports the notion that cathodal current may play a more critical role in wound healing.

Future multicenter randomized controlled trials should clearly define the principles of electrode placement and polarity in the protocol design phase and incorporate them as predefined variables for stratified or subgroup analyses. For instance, comparing configurations such as “the cathode placed at the wound edge/center *vs*. placed on distal healthy skin” under the same equipment and parameters can help assess their impact on healing rates, pain management, infection control, and adverse events. Additionally, consistent documentation of electrode materials, sizes, contact areas, and distances from the wound edge is essential for enabling subsequent systematic reviews and meta-analyses to more accurately evaluate the relative merits of different electrode strategies.

### 6.4 Expansion of indications and combined application with other treatments

Electrical stimulation, as an adjunctive therapy for diabetic foot ulcers, typically requires integration with standard comprehensive treatment, which includes debridement, pressure relief, local and systemic infection control, and blood glucose management. Current clinical studies primarily focus on patients with Wagner grade 2 ulcers, while applications in patients with Wagner grade 3–4 deep infections or exposed bone are notably insufficient. This is partly because Wagner grade 3–4 patients often experience severe deep infections, and relying solely on electrical stimulation combined with local antibiotics may not effectively halt the spread of infection. Moreover, even though electrical stimulation demonstrates some inhibitory effects on common wound pathogens such as Staphylococcus aureus, Pseudomonas aeruginosa, and Escherichia coli, its mechanism mainly depends on the electrolysis of the medium to alter local pH (78). In cases of severe infection, it cannot replace systemic anti-infective treatments.

Patients with Wagner grade 3–4 ulcers generally require thorough debridement combined with skin grafting or flap transfer. Merely adding electrical stimulation to conventional treatment may not significantly alter prognosis. However, considering that electrical stimulation can reconstruct the endogenous electric field within the wound and promote the migration and functional activity of various cells, including keratinocytes (79), fibroblasts (80), and endothelial cells (42), there is a theoretical advantage in enhancing the survival of grafts or flaps and accelerating epithelialization at the wound edges. Future clinical research should focus on the efficacy of electrical stimulation combined with skin transplantation or flap reconstruction, exploring its expansion of indications in high-grade diabetic foot ulcers.

Diabetic foot ulcers are often associated with varying degrees of lower limb ischemia, and improving local blood supply to the wound is a critical factor in promoting healing (81). Neovascularization not only supplies oxygen, nutrients, and various growth factors to the wound but also recruits macrophages and other monocytes to participate in inflammation regulation and tissue repair (82). Studies have shown that electrical stimulation can enhance blood flow perfusion to the wound and promote the expression of factors such as vascular endothelial growth factor (VEGF) (83), thereby contributing to the formation of new blood vessels. Consequently, in ischemic or combined ischemic diabetic foot ulcers, electrical stimulation may yield more significant healing benefits compared to neuropathic patients, warranting further typological analysis in future research.

For neuropathic diabetic foot ulcers primarily characterized by peripheral neuropathy, electrical stimulation as a local treatment modality can be easily combined with peripheral nerve decompression surgery and spinal cord stimulation, thereby forming a synergistic effect in improving microcirculation and alleviating neuropathic pain. By designing subgroup comparisons in future trials based on different pathological types (ischemic, neuropathic, and mixed) and various combined treatment strategies, it is anticipated that the optimal positioning and usage strategies of electrical stimulation for different diabetic foot ulcer phenotypes can be further clarified.

## 7 Conclusion

Existing clinical and experimental studies indicate that electrical stimulation, as an adjunctive therapy for diabetic foot ulcers, has clear potential advantages in promoting wound healing, improving local blood supply, and regulating inflammatory responses. Among various forms of electrical stimulation, self-powered nanogenerators, which convert mechanical energy into electrical energy, significantly reduce reliance on external power sources and wires. This innovation alleviates the inconvenience associated with the need to repeatedly secure electrodes during wearing, movement, and dressing changes, potentially improving patient adherence over the long term. Although related technologies are still in the preclinical stage, advancements in new power supply systems and conductive materials suggest that nanogenerators may become one of the most promising modalities for future electrical stimulation.

In terms of parameter selection, current evidence is insufficient to support a unified “best regimen” applicable to all patients; however, several relatively clear trends can be summarized: under safe and tolerable conditions, high current densities and prolonged exposure to direct current should be avoided. Preference should be given to low-intensity, predominantly cathodal electric field configurations, using high-voltage, low-duty cycle monophasic rectangular pulse currents to balance sufficient directional cues with minimal thermal load on the tissue. Clinical studies commonly adopt treatment regimens of once daily or every other day for 30 to 60 minutes over a duration of four weeks. It is important to emphasize that these parameters and operational guidelines should be viewed as a “relatively preferred strategy” based on the current evidence, which still requires further validation and refinement through prospective, multicenter randomized controlled trials.

To promote standardized and large-scale application of electrical stimulation in the field of diabetic foot ulcers, future multicenter studies should prioritize the following areas: first, during the inclusion and analysis phases, clearly distinguish between ischemic, neuropathic, and mixed diabetic foot ulcers (DFUs) and predefine stratifications for different pathological phenotypes; second, consistently and comprehensively report key stimulation parameters, electrode placement methods, and primary clinical outcomes, facilitating horizontal comparisons and dose-response analyses between different studies; third, systematically evaluate the combined efficacy of electrical stimulation (including wearable nanogenerator systems) with comprehensive strategies such as vascular reconstruction, skin grafting/flap reconstruction, and negative pressure therapy, especially in high-risk populations like those with Wagner grades 3–4.

Currently, there are significant evidence gaps regarding safety data and standardization, including insufficient systematic safety assessments for high-risk groups such as patients with implanted pacemakers or ICDs, severe infections, exposed bone wounds, and suspected malignant ulcers. There is also a lack of proactive and standardized reporting of adverse events and long-term follow-ups, alongside inconsistent recordings of stimulation parameters, electrode configurations, and device performance. Overall, electrical stimulation provides a promising physical intervention option for the comprehensive management of diabetic foot ulcers, but to genuinely integrate it into standardized treatment pathways, rigorously designed multicenter trials are necessary to further clarify the most promising stimulation modalities, optimize parameter combinations, and systematically address key evidence gaps regarding safety and standardization.
